# Seizure burden and neuropsychological outcomes of new-onset refractory status epilepticus: Systematic review

**DOI:** 10.3389/fneur.2023.1095061

**Published:** 2023-01-24

**Authors:** Olga Taraschenko, Spriha Pavuluri, Cynthia M. Schmidt, Yashwanth Reddy Pulluru, Navnika Gupta

**Affiliations:** ^1^Division of Epilepsy, Department of Neurological Sciences, University of Nebraska Medical Center, Omaha, NE, United States; ^2^Leon S. McGoogan Health Sciences Library, University of Nebraska Medical Center, Omaha, NE, United States

**Keywords:** chronic NORSE, febrile infection epilepsy-related syndrome (FIRES), refractory seizures, encephalopathy, cognitive failure, mood disturbances, functional outcomes, seizure outcomes

## Abstract

**Background:**

Long-term sequelae of the new onset refractory status epilepticus (NORSE) include the development of epilepsy, cognitive deficits, and behavioral disturbances. The prevalence of these complications has been previously highlighted in case reports and case series: however, their full scope has not been comprehensively assessed.

**Methods:**

We conducted a systematic review of the literature (PROSPERO ID CRD42022361142) regarding neurological and functional outcomes of NORSE at 30 days or longer following discharge from the hospital. A systematic review protocol was developed using guidance from the Preferred Reporting Items for Systematic Reviews and Meta-Analyses (PRISMA).

**Results:**

Of the 1,602 records for unique publications, 33 reports on adults and 52 reports on children met our inclusion criteria. They contained the description of 280 adults and 587 children of whom only 75.7 and 85% of patients, respectively had data on long-term follow-up. The mean age of adult and pediatric patients was 34.3 and 7.9 years, respectively; and the longest duration of follow up were 11 and 20 years, respectively. Seizure outcomes received major attention and were highlighted for 93.4 and 96.6% of the adult and pediatric NORSE patients, respectively. Seizures remained medically refractory in 41.1% of adults and 57.7% of children, while seizure freedom was achieved in only 26 and 23.3% of these patients, respectively. The long-term cognitive outcome data was provided for just 10.4% of the adult patients. In contrast, cognitive health data were supplied for 68.9% of the described children of whom 31.9% were moderately or severely disabled. Long-term functional outcomes assessed with various standardized scales were reported in 62.2 and 25.5% of the adults and children, respectively with majority of patients not being able to return to a pre-morbid level of functioning. New onset psychiatric disorders were reported in 3.3% of adults and 11.2% of children recovering from NORSE.

**Conclusion:**

These findings concur with previous observations that the majority of adult and pediatric patients continue to experience recurrent seizures and suffer from refractory epilepsy. Moderate to severe cognitive disability, loss of functional independence, and psychiatric disorders represent a hallmark of chronic NORSE signifying the major public health importance of this disorder.

## 1. Introduction

New onset status epilepticus (NORSE) and its subcategory Febrile Infection Related Encephalopathy Syndrome (FIRES) have been described in adult and pediatric patients under various terms starting as early as 1950's. The term NORSE was coined by Wilder-Smith in 2005; the definition and clinical criteria were formalized in 2018 (1–3). NORSE encompasses various clinical presentations of *de novo* recurrent refractory seizures without evidence of acute structural, metabolic, or toxic causes (3). The true incidence of NORSE is unknown; however, it may constitute up to 20% of all cases of refractory status epilepticus (4, 5). NORSE most frequently occurs in previously healthy young adults and school-aged children; however, older individuals, including septuagenarians, have also been affected. In adult case series, a higher prevalence has been reported in women. This contrasts with pediatric case series where NORSE predominantly affects boys (1, 6–9). An etiology is identified in up to 50% of the adult patients with NORSE, most of whom suffer from primary or paraneoplastic autoimmune encephalitis (7). On the other hand, paraneoplastic and autoimmune NORSE is rare in children (10–12).

A prodromal phase has been reported in 60–100% of patients with NORSE. The prodrome precedes the onset of seizures and status epilepticus (SE) by 1–14 days (7–9, 13) and includes fever, a cardinal diagnostic criterion of FIRES, in 34–91% of patients (7–9). Other prodromal symptoms include headache, mild gastrointestinal or upper respiratory illness, and behavioral disturbances (7, 8). Electroencephalogram (EEG) abnormalities are present in all NORSE patients, and seizures are detected in more than 88% of patients undergoing continuous video EEG monitoring (7, 8). Brain magnetic resonance imaging (MRI) as well as laboratory examination of serum and cerebrospinal fluid (CSF) for the presence of autoantibodies or abnormal immunoglobulin indexes are routinely performed and are frequently abnormal (7–9).

Strides have been made in evidence-based care for NORSE patients. Criteria for the diagnosis of NORSE has been introduced and accepted by the neurology community (3). In addition, evidence- and experience-based recommendations for the management of patients with NORSE have been published by experts from the International NORSE Consensus Group (14, 15). While significant progress has been made in delineating the diagnostic and treatment approaches for NORSE, less emphasis has been placed on studying clinical outcomes, including the long-term sequalae, after the hospital discharge. Further, the literature focuses primarily on seizure outcomes, as refractory epilepsy represents the most significant disability in survivors of NORSE (1, 2, 6–12, 16–99). However, over two-thirds of patients experience moderate to severe cognitive disability following hospitalization or remain in a vegetative state (5, 6, 92, 93, 99). Reports concerning functional limitations after NORSE are sparse and include components of formal functional assessment or narrative descriptions of impaired academic performance or activities of daily living (1, 2, 6–12, 16–99). The emergence of psychiatric and behavioral disturbances after NORSE have also been described; however, the full spectrum of these complications have not been systematically assessed outside of the time of hospitalization.

In this study, we conducted a systematic review of literature on NORSE and compiled data on the neurological and psychiatric outcomes of NORSE at 30 or more days following hospitalization. The long-term outcomes of SE and refractory SE have been documented in recent systematic reviews where the patient symptoms were assessed starting as early as 30 days after the discharge (100–102). Consistent with previously set criteria (100–102), we considered to use a 30-day mark as an appropriate interval after which the outcomes of SE were considered “long-term.” Given that some of these complications may improve over time, we did not restrict the length of follow-up after hospitalization in these reports. We disaggregated the findings in adult and pediatric patients and summarized the key demographic and clinical features of these cohorts. The purpose of this systematic review is to tackle the special circumstance of *de-novo* SE and highlight the spectrum of neurocognitive disabilities in patient recovering from NORSE.

## 2. Methods

The systematic review protocol was developed using guidance from the Preferred Reporting Items for Systematic Reviews and Meta-Analyses (PRISMA) statement (103) and registered in the International Prospective Register of Systematic Reviews (PROSPERO, Center for Reviews and Dissemination number CRD42022361142) (104).

### 2.1. Search strategy

Literature searches were initially carried out from May 30–June 1, 2021, and later updated with the final update on October 13, 2022. MEDLINE (EBSCOhost), CINAHL (EBSCOhost), APAPsycINFO (EBSCOhost), EMBASE (embase.com, version including 1974-present), Scopus, and the Cochrane Library (including the Cochrane Database of Systematic Reviews and The Cochrane Central Register of Controlled Trials, wiley.com) were searched from inception to the final search date. The search strategy was developed by a librarian (C.S.) in consultation with epileptologist with research interest in neuroimmunology and clinical subspecialty in autoimmune epilepsy (O.T.). Each database search included terms representing the “long-term follow-up” and “NORSE” concepts (see complete search strategies available at https://digitalcommons.unmc.edu/search/14). The “long-term follow-up” concept was represented by a variety of subject headings and keywords. Since none of the databases we used had a subject heading for the “NORSE” concept, this concept was represented by keywords and key phrases alone. The following alternate names and acronyms for NORSE were considered during search strategy development: new-onset refractory status epilepticus, NORSE, febrile infection-related epilepsy syndrome, FIRES, febrile illness-related epilepsy, fever-induced refractory epileptic encephalopathy, idiopathic catastrophic epileptic encephalopathy, severe refractory status epilepticus owing to presumed encephalitis, devastating epilepsy in school-age children, DESC, acute non-herpetic encephalitis with refractory repetitive partial seizures, acute encephalitis with refractory repetitive partial seizures, AERRPS, *de novo* cryptogenic refractory multifocal febrile status epilepticus. The search strategies were designed to retrieve records containing any of these names listed and to retrieve records containing any of the listed acronyms if the record in question also contained a word beginning with one of the following word trunks: epilep, convul, or seizur. Since no funds were available for translation, English- language filters were applied. Conference abstracts, editorials, and review articles were separated from other search results when filters allowed.

### 2.2. Study selection and quality assessment

**Figure 1:**
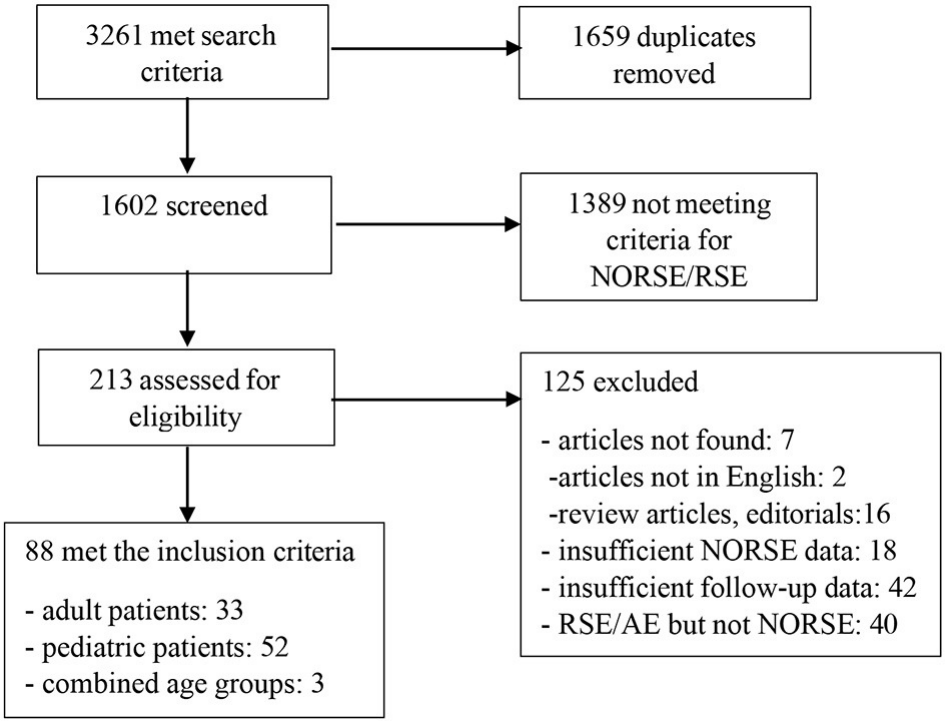
Search strategy flow diagram.

The database searches retrieved 3,261 total records (142 from CINAHL, 24 from the Cochrane Library, 637 from EMBASE, 911 from MEDLINE, 306 from PsycINFO and 1,241 from Scopus, Figure 1). All search results were imported into RefWorks and 1,659 duplicate records were removed using RefWorks' and Zotero duplicate detection tools. A total of 1,602 unique publications remained for title/abstract review (Figure 1). The titles and abstracts were reviewed by two independent neurologists (S.P. and N.G.) for the inclusion criteria and selected articles were chosen for full review. Disagreements between reviewers and inquiries by reviewers were resolved by another reviewer. Reports on other types of SE that did not fulfill the criteria of NORSE as well as those with outcomes reported at <30 days following the discharge were excluded. Case reports and case series were evaluated based on completeness and quality of reporting and were excluded if they did not provide the pertinent information (105).

### 2.3. Data extraction

Data extraction was performed using a standardized template. In addition to basic demographic data, the following outcomes were extracted: duration of follow-up, proportion of patients with seizures controlled with anti-seizure medications (ASM) or other treatments, proportion of patients with refractory seizures and recurrent SE, presence and severity of acquired cognitive disability with specific reference to memory impairment. In addition, functional outcomes assessed with Modified Rankin Scale (mRS), Clinical Global Impressions of Improvement Scale (CGI-I), Pediatric Cerebral Performance Score (PCPS), or Glasgow outcome score (GOS) at the last follow-up visit and proportion of patients with acquired neurological comorbidities, psychiatric complications, and learning disabilities were extracted.

### 2.4. Data analysis

Age was determined through weighted means, when possible. The sex distribution, proportions of patients with seizures, SE, cognitive failure, and other comorbidities were assessed when possible. Comparisons of results between the age groups were descriptive only and not statistically assessed. The summaries were disaggregated for the adult patients (19 years and older), the children, and the mixed age cohort. Given that the age range of patients in the individual reports was broad, the outcomes for various age groups could not be stratified. Similarly, given that the range of follow-up time was broad in the individual reports and these intervals were largely non-overlapping, the outcomes for the specific time intervals were not assessed.

## 3. Results

### 3.1. Demographic patient characteristics

From 1,602 publications identified by the search, 1,389 reports were excluded after the initial title and abstract screening as they did not meet criteria for NORSE/refractory status epilepticus (RSE), and 213 reports were selected for full manuscript review (Figure 1). Of these 213 articles, 33 studies in adults and 52 studies in children met the inclusion criteria (Figure 1). Three reports contained findings for adult and pediatric age groups. Collectively, the reports contained a description of 280 adult and 587 pediatric patients of which the data on the long-term seizure and cognitive outcomes were available for 212 (75.7 %) adult and 499 (85%) pediatric patients, respectively. In two studies that did not disaggregate findings by age (166 patients total), the data on long-term outcomes were available in 127 (76.5%) patients (51, 59).

In the adult group, the mean age of patients was 34.3 years, and the majority (77.6%) were female. The working diagnosis of NORSE was established in 146 (68.9%) patients, FIRES in 9 (4.3%) patients, new onset super refractory SE (NOSRSE) in 17 (8%) patients, RSE and superrefractory SE (SRSE) in 17 (8%) patients, autoimmune anti-NMDA receptor encephalitis in 2 (0.9%) patients, unspecified autoimmune encephalitis in 19 (8.9%) patients, and acute encephalitis with repetitive recurrent partial seizures (AERRPS) or presumed limbic encephalitis in 2 (0.9%) patients. Pertinent laboratory findings in these patients included the autoantibodies against NMDA receptors (28), GABA_A_ receptors (1), GABA_B_ receptors (2), voltage gated potassium channel complex (VGKC, 7), contactin-associated-protein (CASPR,1), type 1 anti-neuronal nuclear protein (ANNA-1, anti-Hu, 1) and GAD-65 proteins (8) in the serum or CSF; however, the tumor status of patients was not consistently recorded. In the large case series on NORSE, paraneoplastic antibodies in patients with confirmed malignancies included anti-Ro (1), anti- NMDA receptor (9), anti-VGKC (3), anti-Hu (3), anti-voltage gated calcium channel (VGCC, 2) antibodies and collapsing response mediator protein 5 (CRMP-5, 1) (5). The identification of infectious agents, including Epstein-Bar virus and other pathogens (Cytomegalovirus, Herpes simplex virus, human immunodeficiency virus, *Mycoplasma pneumonia*e, *Treponema pallidum, Toxoplasma gondii*, Varicella zoster virus, West Nile virus) were reported in 14 patients. Collectively, the etiology of NORSE appeared to be established in 37.7% of 106 patients described in articles meeting our inclusion criteria. Other findings in adult patients were signal abnormalities on the MRI (4) or mass spectroscopy (1) of the brain.

In pediatric studies, the mean age of patients was 7.9 years, and 42.5 % were female. The diagnosis of NORSE or FIRES were established in 149 (29.8%) and 237 (47.4 %) of patients, respectively. Other diagnoses included AERRPS in 35 (7%) patients, devastating encephalopathy in school age children (DESC) in 14 (2.8%) patients, hemiconvulsion-hemiplegia syndrome (HHE) in 35 (7%) patients, SE-related presumed encephalitis in 19 (3.8%) patients, *Mycoplasma pneumoniae* encephalitis in 5 (1%) patients, anti-NMDA receptor encephalitis in 3 (0.6%) patients, as well as anti-GAD encephalitis, steroid-responsive encephalopathy and associated autoimmune thyroiditis (SREAT) or SRSE in 1 patient each (0.2%). The pertinent findings in patients' evaluation included presence of autoantibodies in the serum and CSF specimens in 19 (3.2%) patients. The antibodies against NMDA receptors (2), GABA_A_ receptors (1), AMPA-GluR3 receptors (1), and GAD-65 protein (6) as well as elevated serum and CSF anti-thyroid peroxidase (TPO) antibodies (1) were documented along with the increase of CSF cytokines such as tumor necrosis factor and interleukins 6 and 10 (1). Genetic deficiencies (5) and secondary hemophagocytic lymphohistiocytosis (3) were also recorded as the possible etiologies of NORSE. Brain MRI examinations were abnormal in 31 patients and revealed various degrees of cortical atrophy (10), signal changes in the claustrum (1), temporal cortical regions (2), and cerebellum (1) as well as bilateral hippocampal atrophy (1).

In two large reports that collectively included 127 adult and pediatric patients, the proportion of female patients was smaller than male (41.7%); but the age of participants was not consistently provided (51, 59). The autoimmune antibodies were not identified in serum and CSF of these patients. The cortical and hippocampal signal abnormalities on brain MRI were reported in 27 patients.

### 3.2. Seizure outcomes

The duration of follow-up in the adult group ranged from 30 days to 11 years. Seizure outcomes were reported in 198 out of 212 patients (93.4%, Supplementary Table 1). Of these, 10 (5.1%) patients become seizure-free and discontinued the ASMs, while 28 (14.1%) and 15 (7.6%) patients were seizure-free when receiving the ASMs alone or ASM in combination with other treatments, respectively. In the 10 patients who were seizure-free, the treatment status was not reported. Medically refractory epilepsy was diagnosed in 82 (41.4%) patients for whom other treatment approaches were tried, including immunotherapies [steroids, intravenous immunoglobulin (IVIG), tacrolimus, mycophenolate mofetil, rituximab, anakinra, plasma exchange, cyclophosphamide] in various combinations (25) as well ketogenic diet (5), modified Atkin's diet (3), neurostimulation (2) and focal cortical resections (5). Delayed recurrent SE was reported in two patients.

In the pediatric group, the duration of follow-up ranged from 30 days to 20 years. Seizure outcomes were reported for 482 (96.6%) patients (Supplementary Table 2). Forty-seven patients (9.8%) were seizure free without ASMs, while 66 (13.7%) patients achieved seizure control with ASMs alone or when treated with ASMs combined with ketogenic diet. The treatment status in an additional 27 patients was not recorded, although they were reported to be seizure free. Medically refractory epilepsy was documented in 278 (57.7%) patients of whom 34 patients required therapies beyond ASMs, including steroids (3), IVIG (5), tocilizumab (2), anakinra (2), and ketogenic diet (12). Neuromodulation (7) and focal resections (2) were also listed among the treatments for refractory seizures. Recurrent SE was reported in 8 patients.

### 3.3. Cognitive outcomes

Cognitive outcomes at 30 days or later following the hospital discharge were reported for 22 (10.4%) of the adult patients included in the reviewed literature; most of these patients were described in case reports (Supplementary Table 1). Most of the studies assessed cognitive outcomes subjectively. All available data on neuropsychological testing, including the formal test scores are provided in Supplementary Tables 2, 3. At the last follow-up, 3 patients (13.6%) had normal cognition, 2 (9.1%) had mild cognitive impairment, and 4 (18.2%) and 2 (9.1%) had moderate or severe degrees of cognitive impairment, respectively. Three patients (13.6%) remained in a vegetative state; however, the time of assessment (10 months) was only documented for one patient. Memory impairment was reported in 12 patients (54.5%). Four (33.3%) of these had moderate or severe impairment. Specific reference to working or visual memory impairments were made in 2 patients. Mild naming deficits (1) and persistent impairment of processing speed and verbal memory function (1) were also reported at the last follow-up.

Reports concerning pediatric patients elaborated on cognitive outcomes after hospital discharge more frequently than reports concerning adults. The assessment of cognitive status was documented in 68.9% of all 344 included pediatric patients with NORSE (Supplementary Tables 2, 3). Seventy-eight patients (22.7%) have experienced complete cognitive recovery. Mild impairment was diagnosed in 42 children (12.2%), while moderate and severe loss were noted in 60 (17.4%) and 50 (14.5%) of children, respectively. Thirty patients (8.7%) have remained in a vegetative state. The degree of intellectual disability was not specified in 94 (27.3%) children. References to specific memory impairment were made in reports concerning 19 patients of whom 8 had a severe memory loss. Delayed motor, social, and verbal development were noted in 1 child described in a case report.

### 3.4. Functional outcomes and activities of daily living

In adult reports, mRS scores and activities of daily living data were included in 123 (58%) and 13 (6.1%) of patients, respectively (Supplementary Table 1). In 37 (30%) patients, the specific values of mRS were provided and were as follows: 23 patients (30.1 %) with scores of 0–1, 27 (22.5%) with scores of 2–3, and 24 (19.5%) with scores of 4–6. Eight patients had no formal assessment but were noted to return to baseline or had good recovery and have remained autonomous in their day-to-day functioning. Three patients had not regained consciousness. The GOS was reported in 9 (4.2%) patients of whom 2, 1, and 6 had scores of 5, 3, and 1, respectively (Supplementary Table 1). A report of 14 patients had only narrative characterization of the activities of daily living. Eight of these 14 patients were described as being independent and 6 patients as needing assistance. Other assessments included mentioning of a patient's ability to resume previous academic activities (1) and another patient's referral to a supervised nursing facility (1).

Pediatric patients' functional outcomes as assessed with mRS, PCPS, CGI-I, pediatric GOS, and GOS were reported in 61 (12.2%), 63 (12.6%), 5 (1%), 18 (3.6%), and 16 (3.2%) patients, respectively (Supplementary Table 2). There were 6, 2, and 6 patients with the mRS scores of 0–1, 2–3, and 4–6, respectively. Sixty-three patients were assessed with PCPS. Favorable outcomes, defined as score ≤ 2, were noted in 12 (19.1%) children while unfavorable outcomes (i.e., score >3) were noted in 51 (80.9%) patients. The outcomes assessed with CGI-I were distributed as follows: favorable (i.e., score of 2–3) in 3 patients, and without change (i.e., score 4) in 2 patients. The subdomains of communication and autonomy were reported in 5 patients. Pediatric GOS was used in two studies to assess outcomes with a score of 1 and 4 corresponding to the good recovery and vegetative state, respectively. There were 4, 3, 7, and 4 patients with scores of 1, 2, 3, and 4, respectively. Three other studies utilized GOS with scores that were defined differently such that 5 was consistent with good recovery and 2 was consistent with vegetative state. Scores of 5, 4, 3, and 2 were reported in 7, 4, 1, and 4 patients, respectively. Of 87 patients, for whom the activities of daily living were characterized, 55 (63.2%) were independent and 32 (36.8%) required assistance. In the narrative descriptions of the functional status, there were reports of resuming premorbid academic activities (3), having some academic difficulties (14), requiring special education (1), developing learning disabilities (10), having attention deficits and executive dysfunction (2), and developing severe developmental delay (5).

### 3.5. Acquired psychiatric comorbidities and neurological deficits

The emergence of new onset psychiatric disorders in adult survivors of NORSE were reported in only 7 (3.3%) patients (Supplementary Table 1). These manifestations included schizophrenia (1), attention deficit disorder (2), Capgras syndrome (1), psychomotor agitation (1), and personality changes (2). Various neurological deficits were reported in 6 patients and included bilateral lower extremity weakness and mild ataxia (1), right hemiparesis and dysphasia (1), mild receptive dysphasia (1), and moderate language impairment (1) as well as unspecified gait disturbance (1).

The description of psychiatric complications of NORSE were more common in the pediatric literature and were provided for 56 (11.2%) of all included children (Supplementary Table 2). Reports concerning 56 of the pediatric patients mentioned behavioral disturbances of various severity: 13 developed aggression, 2 suffered from emotional lability, 4 had apathy, and 1 had conduct disorder. Mild or severe attention deficit hyperactivity disorder and various manifestations of attention impairment were diagnosed in 9 patients and an autism spectrum disorder was reported in 1 patient. One child attempted suicide. Various neurological sequalae of NORSE were described in 108 (21.6%) children. Specifically, the long-term motor deficits included hemiplegia (35, 32.4%), unspecified motor impairments (36, 33.3%) and unilateral tongue weakness (1, 0.01%). Other impairments included visual field deficits (1), peripheral neuropathy (2), ataxia (3), choreoathetoid movements (1), and tremor (3). Language deficits were reported in 20 (18.5%) patients. One of these children improved after the initiation of responsive neurostimulation.

## 4. Discussion

In the present study, we systematically reviewed and summarized the literature on the long-term neurological and psychiatric outcomes of NORSE in adult and pediatric patients who survived longer than 30 days after hospital discharge. We found that seizure status is assessed in over 90% of patients who had the data on the long-term outcomes. However, the cognitive outcomes were only included in one tenth of these reports in adults and nearly two-thirds of the reports concerning children. Functional outcomes were included in more than 60% of studies of adults and more than 25 % of studies of children. Unfortunately, the functional outcomes were measured using four different outcome scales limiting our ability to synthesize study results into an understanding of the overall scope of associated disability. New onset psychiatric disorders were under-reported and were only included in a small proportion of the reports. Overall, these findings reflect the lack of standardization for the reporting of outcomes, particularly for reporting symptoms other than seizures, and may also reflect a gap in the care of these patients after the initial hospital encounters.

### 4.1. Seizure outcomes

Consistent with previous observations, in this systematic review, we found that majority of adults and children with NORSE will continue to have seizures 30 days or later after the hospital discharge. Of those who continue to have seizures, 41.1% of adults and 57.7% of children will remain refractory to either conventional ASDs used alone or in combination with immunotherapies, ketogenic diet, and neurostimulation. The pathogenesis of recurrent seizures in NORSE is not clear. Several proposed mechanisms of uncontrolled seizure generation during the acute phase of NORSE included aberrant signaling in the interleukin (IL)-1 and toll-like receptor (TLR)-mediated pathways, overactivation of the NLRP3 inflammasome as well as functional or genetic deficiency of IL-1 receptor antagonist activity (48, 67, 106–113). These mechanisms can also be involved in late seizure recurrence in survivors of NORSE. Of note, chronic epilepsy in cryptogenic NORSE develops without a latent period which is distinct from the post-infectious epilepsies associated with viral or bacterial pathogens (10, 114).

### 4.2. Cognitive outcomes

Our findings add to those of previous studies that showed only a small proportion of patients have achieved their pre-morbid cognitive function after they were discharged from the hospital (9). Nearly one-third of adults or children continue to suffer from moderate and severe intellectual disability. In a case series of 14 pediatric patients with NORSE, all children attended special education in the later course, and seven patients had severe cognitive failure. The primary impairment involved deficits in frontal lobe function and was manifesting as the lack of motor and speech initiative, major slowness, perseveration, and poor attention (9, 10). While there were no specific patterns in neuropathological findings in NORSE, gliosis, laminar cortical necrosis, and diffuse cortical atrophy are the shared common features (1, 6, 99, 115, 116). Moreover, various degrees of persistent inflammation and structural changes such as mesial temporal sclerosis may contribute to the severity of cognitive phenotype.

Chronic cognitive disability after NORSE represents the major public health problem. Since many patients are previously healthy, severe cognitive impairment or vegetative states after NORSE are devastating. A need exists for comprehensive chronic care that includes cognitive rehabilitation for patients and respite for caregivers. The mechanism of cognitive failure in NORSE is unclear, but it is likely linked to the severity and duration of seizures (91). In subtypes of autoimmune encephalitis that can manifest as NORSE (e.g., anti-NMDA receptor encephalitis), antibodies were found to be directly pathogenic for memory failure and seizures. However, the pathogenesis of epileptic encephalopathy in other antibody-mediated or cryptogenic NORSE is not clear (7, 117, 118). The lack of the uniformed objective measurements of cognitive function noted in the reviewed literature represents a major shortcoming of this research. More systematic and comprehensive objective testing of survivals is needed in future studies.

### 4.3. Functional outcomes and activities of daily living

We found that a comprehensive approach in documenting the functional outcomes in survivors of NORSE were lacking. Further, only one-fourth of the children and 60% of adults in our study had their functional outcomes assessed and reported. While the mRS was the most common outcome scale applied in adult patients, there were three additional outcomes scales used in pediatric patients. Such inconsistent data availability in outcomes particularly for the pediatric patients introduce a potential bias and limits definitive conclusions. In the recent systematic analysis of functional outcomes in autoimmune encephalitis, it was established that mRS had poor sensitivity for cognitive disability and mood disturbances in encephalitis at follow-up (119). There was an additional focus on the academic performance and other aspects of social functioning in children, which were consistently underreported in the adult literature. Given that many patients with NORSE have now survived for several decades, a more standardized approach in categorization of their functional abilities is needed to monitor their recovery and develop guidelines for individualized rehabilitation.

### 4.4. Acquired psychiatric comorbidities and neurological deficits

Psychiatric comorbidities, including recurrent psychosis are frequently encountered in severe epileptic encephalopathies (120); however, the prevalence of mental illness in association with recent refractory SE is unknown. Multiple factors could contribute to the development of psychosis and depression in patients recovering from NORSE, including the individual vulnerability to the effects of ASDs or immunotherapy as well as prolonged brain hypoxia (121). The presence of specific autoimmune antibodies (such as anti-AMPA or anti-NMDA receptor antibodies) in NORSE with established etiologies can guide the anticipation of chronic psychiatric sequalae (122). The accounts of new onset psychiatric disorders in only 3.3 and 11.2% of the adults and pediatric patients with chronic NORSE likely reflects underreporting and insufficient attention to these comorbidities on the part of neurologists involved in care of these patients.

### 4.5. Limitations

Our study has several limitations. We found that the majority of reports on long-term outcomes of NORSE are focused on seizure status while other domains of the neurological and psychiatric health were assessed and commented on inconsistently. This likely represents a reporting bias and underreporting in various relevant health domains for the most severely disabled patients (e.g., those in vegetative state) and those whose seizures were under better control. This limits our conclusions regarding the prevalence of multiple manifestations of chronic NORSE other than seizures. Given the retrospective and observational design of most reviewed studies, it is unclear whether the severity of SE and its refractoriness can be linked to any of the reported outcomes. This impedes our progress in understanding the mechanisms of cognitive failure and development of psychiatric comorbidities in NORSE. Given that the analysis in specific age categories in pediatric patients was not feasible, the outcomes of SE at different stages of brain development and the effects of age-related compensatory abilities have not been accounted for. Likewise, the inability to disaggregate the data into the specific duration of follow-up precluded the analysis of outcomes at different stages of recovery from NORSE. Lastly, we acknowledge the selection and information bias that was not specifically assessed in this systematic review.

## 5. Conclusions

We found that most patients with chronic NORSE continue to experience recurrent seizures, and seizure treatment and reporting remain the main focus of the literature. Documentation of cognitive disability, loss of functional independence, and onset psychiatric manifestations have been inconsistent and should be interpreted with caution given the methodological limitations. While challenging to implement due to the rarity and geographic dispersion of NORSE, future prospective studies may help provide high-quality evidence to guide the management and rehabilitation of these patients.

## Author contributions

OT: conceptualizing the study, drafting of the manuscript for content, and major role in the analysis and interpretation of data. SP: main role in the acquisition and analysis of data. CS: main role in the acquisition and analysis of data as well as study design. YP: acquisition and analysis of data. NG: drafting of the manuscript and main role in the acquisition and analysis of data as well as study design. All authors contributed to the article and approved the submitted version.
