# Supplementary material for: Seizure burden and neuropsychological outcomes of new-onset refractory status epilepticus: Systematic review
Source: Front Neurol. 2023 Jan 24;14:1095061. doi: 10.3389/fneur.2023.1095061 (PMC9902772; doi:10.3389/fneur.2023.1095061)
Supplement: Supplementary file 1 [file Table_1.DOCX]

| **Supplemental table 1: Long-term outcomes in adult patients with NORSE** | | | | | | | | | | | |
| --- | --- | --- | --- | --- | --- | --- | --- | --- | --- | --- | --- |
| **Study** | **Number of patients (outcome data available)** | **Age or age range (years)** | **Follow-up (months)** | **Outcomes** | | | | | | |  |
|  |  |  |  | **Seizure-outcomes (% patients with long-term outcomes)** | | **Cognitive impairment (% patients with long-term outcomes)** | | | **Functional outcomes (% patients with long-term outcomes)** | **Psychiatric and behavioral issues (% patients with long-term outcomes)** |  |
|  |  |  |  | **Seizure-free^#^ or controlled with ASM, other treatments** | **Refractory seizures (ASM, other treatments)** | **None** | **Mild** | **Moderate/ severe** |  |  |  |
| Acampora (2022) ^16^ | 1 | 23 | 1-12 |  |  |  |  |  |  |  |  |
| Aydemir (2022) ^19^ | 1 | 27 | 2 |  | 100 | 100 |  |  |  |  |  |
| Choi (2021) ^29^ | 1 | 38 | > 1 |  | 100 |  |  |  |  |  |  |
| Donnelly (2021) ^30^ | 1 | 26 | 2 |  |  |  | 100 |  |  |  |  |
| Gugger (2020) ^37^ | 20 (15) | NA | 2 - 42 |  | 100 |  |  |  | mRS 0-2: 53.3 |  |  |
| Kim (2020) ^40^ | 39 (34) | 19-63 | 6 | 11.8^#^, 41.2 | 47.1 |  |  |  |  |  |  |
| Matthews (2020) ^41^ | 26 (17) | NA | 2-22 |  |  |  |  |  | mRS 0-3: 71; mRS 4-5: 29 |  |  |
| Al-Khateeb (2019) ^44^ | 1 | 41 | 7 | 100 |  |  |  | 100 |  | schizophrenia |  |
| Fatuzzo (2019) ^45^ | 1 | 29 | 24 |  | 100 |  |  |  |  | attention deficit |  |
| Basha (2017) ^61^ | 9 (3) | 20-56 | 18-84 |  | 33.3 |  |  |  |  |  |  |
| Dillien (2016) ^65^ | 1 | 27 | 6 |  | 100 |  | 100% |  |  | attention deficit |  |
| Gagnon (2016) ^66^ | 1 | 38 | 21 | 100 |  |  |  |  | autonomous |  |  |
| Gaspard (2015) ^7^ | 97 (63) | NA | 6-8 | 8^#^ | 37 |  |  |  | mRS: 0-1: 41; mRS 2-3: 38  mRS: 4-5: 16; mRS 6: 5 |  |  |
| Hainsworth (2014) ^74^ | 1 | 24 | 6 | 100 |  |  |  |  |  |  |  |
| Marques (2014) ^76^ | 1 | 30 | 12 |  |  |  |  |  |  | aggression/ restlessness |  |
| Juhász (2013) ^81^ | 1 | 56 | 36 |  |  |  |  |  |  |  |  |
| Kumar (2013) ^82^ | 1 | 30 | 36 |  | 100 |  |  |  |  |  |  |
| Gordon Boyd (2012)^85^ | 1 | 22 | 5 |  | 100 |  |  | 100 | resumed work part-time |  |  |
| Johnson (2010) ^90^ | 1 | 35 | 6 |  |  |  |  |  | no functional limitations |  |  |
| Costello (2009) ^6^ | 6 (5) | 24-36 | 18 – 132 | 20^#^, 20 | 60 | 20 |  | 40 (40 had severe) | resumed employment: 80; did not resume employment: 20 |  |  |
| Westbrook (2019) ^48^ | 1 | 21 | 6 | 100 |  | 100 |  |  | returned to baseline |  |  |
| Wilder-Smith (2005)^1^ | 7 (2) | 20-52 | >1 |  | 100 |  |  |  | vegetative state |  |  |
| Steriade (2018) ^58^ | 19 | NA | 1.44 - 26.4 | 58 | 42 |  |  |  | mRS ≥ 2: 47 |  |  |
| Matsuzono (2014) ^77^ | 1 | 22 | 10.3 | 100 |  |  |  |  |  |  |  |
| Verma (2013) ^84^ | 1 | 35 | >1 | 100 |  |  |  |  |  |  |  |
| Patel (2017) ^64^ | 1 | 19 | 10 |  | 100 |  |  |  | vegetative state |  |  |
| Tian (2014) ^72^ | 98; 12 had SRSE (6) | 19-81 | 3-6 |  |  |  |  |  | *GOS: 5: 50; GOS 4: 16.7; GOS 2: 16.7; GOS 1: 16.7 |  |  |
| Acar (2021) ^27^ | 1 | 62 | 6 |  |  |  |  |  |  |  |  |
| Karunaratne (2021) ^32^ | 1 | 45 | 12 |  |  |  |  |  |  | Capgras syndrome, pseudohallucination |  |
| Wang (2021) ^35^ | 13 (11) | NA | 8-37.5 | 100 |  |  |  |  | mRS < 2, 3 at 18.2 months;  mRS < 2, 6 at 80 months;  mRS < 2, 9 at 100 months | personality change: 18.2 |  |
| Al-Chalabi (2022) ^17^ | 1 | 47 | 3 |  |  |  |  |  | mRS 5 (comatose) |  |  |
| Nawfal (2021) ^23^ | 1 | 21 | 3 |  |  |  |  |  |  |  |  |
| Obara (2022) ^24^ | 1 | 21 | 1-12 |  | 100 |  |  |  |  |  |  |
| ^ Iizuka (2017) ^8^ | 43; 9 had NORSE (9) | 19-59 | 2 |  | 75 |  |  |  | mRS: 1 11.1; mRS 2:11.1; mRS 3: 22.2; mRS 4-5: 44.4; mRS 6:11.1 |  |  |

NORSE, new-onset refractory status epilepticus; ASM, anti-seizure medications; GOS, Glasgow Outcome Score; mRS, modified Rankin scale; SRSE, super-refractory status epilepticus

^#^ Proportion of seizure-free patients

^*^ Legend for GOS: 1: died; 2: not improved; 3-4: improved; 5: recovered to baseline

^^^ Study had individual data for adult and pediatric patients that were reported in the corresponding text
