# Supplementary material for: Seizure burden and neuropsychological outcomes of new-onset refractory status epilepticus: Systematic review
Source: Front Neurol. 2023 Jan 24;14:1095061. doi: 10.3389/fneur.2023.1095061 (PMC9902772; doi:10.3389/fneur.2023.1095061)
Supplement: Supplementary file 2 [file Table_2.DOCX]

| **Supplemental table 2: Long-term outcomes in pediatric patients with NORSE** | | | | | | | | | | | |
| --- | --- | --- | --- | --- | --- | --- | --- | --- | --- | --- | --- |
| **Study** | **Number of patients (outcome data available)** | **Age or age range (years)** | **Follow-up (months)** | **Outcomes** | | | | | | | |
|  |  |  |  | **Seizure-outcomes (% patients with long-term outcomes)** | | **Cognitive impairment (% patients with long-term outcomes)** | | | | **Functional outcomes (% patients with long-term outcomes)** | **Psychiatric and behavioral outcomes (% patients with long-term outcomes)** |
|  |  |  |  | **Seizure free^#^ or controlled with ASM, other treatments** | **Refractory seizures with ASM, other treatments** | **None** | **Mild** | **Moderate** | **Severe** |  |  |
| Aledo-Serrano (2022) ^18^ | 5 | 2 - 18 | 7-20 |  | 100 |  |  |  | 40 | ^&^CGI: CGI-I: 2: 60; 4: 40; CGI-C: 2: 60; 3: 40;  CGI-A: 3: 40; 4: 60;  CGI-B: 2: 40; 3: 40; 4: 20 | behavioral issues: 100 |
| Nath (2021) ^33^ | 1 | 3 | 7 |  | 100 |  |  |  |  |  |  |
| Husari (2020) ^38^ | 40 (31) | NA | 15-86 | 25.8 | 38.7 |  |  |  | 16.10 | mRS 0-3: 87.1, mRS 4-5: 12.9 | behavioral issues: 41.9 |
| Jaafar (2020) ^39^ | 1 | 8 | 1 | 100 |  | 100 |  |  |  |  |  |
| Lam (2019) ^46^ | 25 (20) | 1.6 - 17.2 | 9 -180 | 15 | 85 | 38.9 | 38.9 | 11.1 | 11.1 | vegetative; mRS<6mo: 0: 15; 1-3: 50; 4-6: 35; mRS> 6mo: 0: 27.8; 1-3: 44.4; 4-6: 27.8 | ADHD: 5.6;  emotional lability: 5.6 |
| Albakaye (2018) ^49^ | 35 | 0.3 - 12 | 120 | 8.6 | 91.4 |  |  |  |  |  | suicide attempt: 2.9 |
| Farias-Moeller (2018) ^52^ | 5 | 4-16 | 6 |  | 100 |  | 40 | 20 | 40 | G-tube/tracheostomy-dependent: 40 |  |
| Jafarpour (2018) ^53^ | 79; 4 had NORSE (3) | NA | 21-97 |  | 66.7 |  |  |  |  |  | autism spectrum disorder: 33.3 |
| Lee (2018) ^54^ | 29 (23) | 1.2 - 17.8 | 6 - 156 | 13 | 87 | 26 |  | 26 | 30.4 |  |  |
| Lin (2018) ^55^ | 63 (44) | 0.2 - 18 | 6 | 29.6 | 70.5 |  |  |  |  | ^***^PCPCS: 1: 3.2; 2: 15.9; 3: 34.9; 4: 12.7; 5: 1.6; 6: 6.4 |  |
| Alparslan (2017) ^60^ | 1 | 8 | 24 | 100 |  |  | 100 |  |  |  |  |
| Caputo (2017) ^50^ | 1 | 13 | 3-6 |  | 100 |  | 100 |  |  |  | anxiety/depression |
| Gofshteyn (2016) ^62^ | 7 (5) | 3.9 – 8.5 | 1 -12 | 20 | 80 |  |  |  |  |  |  |
| Kenney-Jung (2016) ^67^ | 1 | 2.8 | 12 |  | 100 | 100 |  |  |  |  |  |
| Capizzi (2015) ^71^ | 1 | 15 | 18 |  | 100 |  |  | 100 |  |  | emotional instability, psychomotor deterioration |
| Barros (2014) ^73^ | 1 | 7 | 24 |  | 100 |  |  |  |  |  |  |
| Hilberath (2014) ^75^ | 1 | 13 | 3-13 |  |  | 100 |  |  |  |  |  |
| Caraballo (2013) ^11^ | 12 | 2-13.5 | 12 - 180 | 16.7 | 83.3 |  |  |  |  |  | behavioral issues: 91.7 |
| Finné Lenoir (2013) ^80^ | 1 | 17 | 12 |  |  |  |  |  |  |  |  |
| Lin (2012) ^87^ | 2 | 4.5 - 10.5 | 3 |  |  |  | 100 |  |  | ^**^GOS`: 4: 100 |  |
| Lin (2012) ^88^ | 6;3 had RSE (2) | 1.5 – 13.5 | 7.3-22.3 | 33.3 | 33.3 |  |  |  |  | ^**^GOS`: 3: 33.3; 5: 33.3 |  |
| Howell (2011) ^86^ | 7 (6) | 8.3-12.2 | 60 - 204 |  | 100 |  | 33.3 | 33.3 | 33.3 |  | normal behavior: 33.3; mild (33.3) or severe (33.3) behavioral issues: |
| Ismail (2011) ^2^ | 1 | 14 | 12 | 100 |  | 100 |  |  |  |  |  |
| Kramer (2011) ^9^ | 77 (68) | 2-17 | > 1 | 2.9^#^, 4.4 | 92.6 | 17.7 | 14.7 | 23.5 | 11.8 |  | attention deficit disorder: number NA |
| Lin (2010) ^91^ | 9; 6 had RSE (5) | 4.8-10.1 |  |  | 100 |  |  |  |  | ^*^p-GOS:2: 20; 3: 40; 4: 40 |  |
| Lin (2009) ^94^ | 9 (7) | 5-15 | 16-61 |  | 100 |  | 28.6 | 14.3 | 28.6 |  |  |
| Lin (2008) ^95^ | 46; 20 had RSE (13) | 0.7 - 16 | 6 | 23.1 | 76.9 |  | 23.1 | 46.2 |  | ^*^p-GOS: 1: 30.8; 2: 15.4; 3: 38.5; 4: 15.4 |  |
| Kramer (2005) ^98^ | 8 (6) | 2.5 - 15 | > 1 |  | 66.7 | 16.7 | 16.7 | 16.7 | 50 |  | severe ADHD: 50 |
| Rivas-Coppola (2016) ^68^ | 7 | 0.25 -9 | 12 |  | 100 |  |  |  | 14 |  |  |
| Saito (2006) ^97^ | 3 | 7 - 10 | 12 - 120 | 66.7 | 33.3 |  | 33 | 33 | 33 |  |  |
| Sato (2016) ^69^ | 1 | 11 | 60 |  | 100 |  |  |  |  |  |  |
| Shyu (2007) ^96^ | 14 (11) | 5.4 – 15.5 | 6 | 9.1^#^ | 90.9 |  |  | 63.7 | 36.4 |  |  |
| Okumura (2009) ^89^ | 2 | 7 -8 | 1 - 13 |  | 100 | 50 |  |  |  |  | mild conduct disorder/oppositional behavior: 50 |
| Singh (2014) ^78^ | 2 | 7 - 10 | 18-20 | 100 |  |  |  | 100 |  |  | ADHD: 50 |
| Patil (2016) ^12^ | 15 (12) | 3 - 15 | 4 - 60 |  | 100 |  | 33.3 | 41.7 | 25 | ^**^GOS`: 5: 5; 4: 16.7; 2: 33.3 | hyperactive: 33.5; behavioral issues: 25 |
| Uchida (2016) ^70^ | 1 | 9 | 24 |  | 100 |  | 100 |  |  |  | attention impairment/social disorder |
| Stredny (2020) ^42^ | 1 | 6 | 12 |  | 100 |  | 100 |  |  |  | attention impairment /behavioral issues |
| Theroux (2020) ^43^ | 1 | 11 | 16 |  | 100 |  |  |  |  |  | ADHD |
| Morita (2017) ^63^ | 1 | 14 | 48 |  | 100 |  | 100 |  |  |  |  |
| Mikaeloff (2006) ^10^ | 14 | 4-11 | 12 - 84 |  |  |  |  | 50 | 50 |  | aggression/agitation: 71.4; apathy/withdrawal: 28.6 |
| Ueda (2014) ^79^ | 6 | 8 - 18 | 12 - 240 | 50 | 50 |  | 50 | 25 | 25 |  | aggression/agitation: 50 |
| Okanishi (2017) ^56^ | 1 | 5 | 50 | 100 |  |  | 100 |  |  |  | ADHD |
| Peng (2019) ^47^ | 7 | 1.5 - 13 | 6-40 |  | 71.4 |  |  |  |  | mRS: 0: 42.9; 1: 14.3; 3: 28.6; 4: 14.3 |  |
| Basso (2022) ^20^ | 1 | 5 | 1 - 60 | 100 |  | 100 |  |  |  |  |  |
| Jain (2022) ^21^ | 7 | 4 -15 | 4-19 | 14.3 | 85.7 |  |  |  |  |  |  |
| Sivathanu (2022) ^26^ | 1 | 7 | 3-12 | 100 |  | 100 |  |  |  | mRS 1 |  |
| Luo (2021) ^22^ | 1 | 2.4 | 3 | 100 |  |  |  |  |  | delayed motor, social and verbal development | ` |
| Perulli (2022) ^25^ | 1 | 11 | 13 | 100 |  |  |  | 100 |  |  |  |
| Baba (2021) ^28^ | 1 | 8 | 12 | 100 |  |  |  |  |  |  |  |
| Horino (2021) ^31^ | 6 (1) | 4 - 8 | 17 |  |  |  |  |  |  |  |  |
| Sculier (2021) ^34^ | 46 (41) | NA | 5 - 60 |  |  | 53.7 |  |  |  |  |  |
| Wu (2021) ^36^ | 92 (71) | NA | 6 | 62^#^, 16.9 | 21.1 | 32.4 |  |  |  | vegetative state: 16.9 |  |
| ^^^Iizuka (2017) ^8^ | 43; 2 had NORSE (2) | NA | 6-111 |  | 100 |  |  |  |  | mRS: 0: 50; 5: 50 |  |

NORSE, new-onset refractory status epilepticus; ADHD, attention-deficit hyperactivity disorder; ASM, anti-seizure medications; CGI-I, Clinical Global Impressions of Improvement Scale; CGI-C, CGI-communication; CGI-A, CGI-autonomy; CGI-B, CGI-behavior; p-GOS, pediatric Glasgow Outcome Score; GOS`, Glasgow Outcome Score; G-tube, gastrostomy tube; mo, months; mRS, modified Rankin scale; NA, not available; PCPCS, Pediatric Cerebral Performance Category Scale; RSE, refractory status epilepticus

^#^ Percentage of seizure-free patients

^*^ Legend for p-GOS: 1: good recovery; 2: moderate disability; 3: severe disability; 4: persistent vegetative state

^**^ Legend for GOS`: 1: dead; 2: vegetative state; 3: severe disability; 4: moderate disability; 5: good recovery

^***^ Legend for PCPCS: 1: normal; 2: mild disability; 3: moderate disability; 4: severe disability; 5: coma/vegetative state; 6: brain death; denominator is 63

^&^ Legend for CGI: 1: very much improved; 2: much improved; 3: minimally improved; 4: no change from baseline; 5: minimally worse: 6: much worse; 7: very much worse

^^^ Study had individual data for adult and pediatric patients that were reported in the corresponding text
