# Supplementary material for: Seizure burden and neuropsychological outcomes of new-onset refractory status epilepticus: Systematic review
Source: Front Neurol. 2023 Jan 24;14:1095061. doi: 10.3389/fneur.2023.1095061 (PMC9902772; doi:10.3389/fneur.2023.1095061)
Supplement: Supplementary file 3 [file Table_3.DOCX]

| **Supplemental Table 3: Results of neuropsychological evaluation** | |
| --- | --- |
| **Study** | **Results of neuropsychological testing** |
| Obara (2022) ^24^ | WAIS-IV showed an improvement in perceptual reasoning with full-scale IQ of 67. WMS-R showed improvement in visual memory. There was no improvement in processing speed and verbal memory. |
| Basso (2022) ^20^ | The testing revealed moderate expressive aphasia and mild receptive aphasia with normal general cognitive abilities. |
| Baba (2021) ^28^ | **Full-scale IQ (measured using WISC) at 1 year follow-up was 54, and patient was transferred to a class for special-need children. |
| Gordon Boyd (2012) ^85^ | The results were consistent with moderate-to-severe impairment in verbal and visual memory, low average-average IQ, and moderately impaired language functioning. The effects of medications could not be excluded. |
| Caputo (2017) ^50^ | The evaluation performed at 6 months showed improvement in executive function and mild impairment in working memory. |
| Caraballo (2013) ^11^ | The data were consistent with normal findings or mental retardation. |
| Howell (2011) ^86^ | The testing revealed intellectual disability ranging from borderline to severe as well as language and memory deficits, behavioral disturbances, attention deficits and processing deficits. |
| Lam (2019) ^46^ | Neuropsychological tests revealed normal cognitive function or intellectual disability (mild, moderate, severe). |
| Lee (2018) ^54^ | ** The IQ testing (WISC-R) was reported to be as normal or consistent with moderate to severe mental retardation and vegetative state. |
| Marques (2014) ^76^ | The assessment at 1 year showed moderate frontal lobe dysfunction characterized by apathy, impulsivity and dysexecutive syndrome. |
| Perulli (2022) ^25^ | Testing revealed moderate intellectual disability with a milder impact on verbal functioning (WISC-IV verbal comprehension 56, perceptual reasoning 48 working memory 42 processing speed 47, total IQ 40). |
| Mikaeloff (2006) ^10^ | The data were reported as verbal IQ. The testing revealed mental retardation, memory difficulties, language disorders, frontal-temporal lobe dysfunction |
| Okanishi (2017) ^56^ | ** The IQ of 90 (WISC-IV) was reported at 32-month follow-up. At 50-month follow-up, patient had mild visuospatial memory impairment and had scored 2636 (at <1 percentile) on Rey-Osterrieth Complex Figure Test |
| Singh (2014) ^78^ | Patient 1 demonstrated moderate impairments in working memory domain, but had average ability to recall auditory verbal narratives and normal fine-motor speed and dexterity (full scale IQ 71). Patient 2 showed impaired phonics and fluency, moderate-to-severe impairment of the processing speed and recall of auditory verbal narratives (full scale IQ 62). |
| Theroux (2020) ^43^ | The testing at 16-month follow-up showed difficulty with memory and attention. Repeat testing at 22-months showed improvement across most cognitive domains, particularly with regards to working memory, processing speed, and memory retention. |
| Stredny (2020) ^42^ | ** IQ subsets for processing speed performance, and verbal IQ were 60, 86, and 81. |

Abbreviations: IQ, Intelligence Quotient; RNS, Responsive Neurostimulator; WAIS-IV, Wechsler Adult Intelligence Scale-Fourth Edition (WAIS-IV); WISC, Wechsler intelligence scale for children; WISC-R, Wechsler Intelligence Scale for Children-Revised; WMS-R, Wechsler Memory Scale-Revised

** - Certain measures of cognitive function were available. It was not clear if patient had a full neuropsychological assessment.
